# MALIGNANT MELANOMA WITH A SEBORRHEIC KERATOSIS-LIKE CLINICAL PRESENTATION

**DOI:** 10.4103/0019-5154.57623

**Published:** 2009

**Authors:** Kunitaka Haruna, Yasushi Suga, Yuki Mizuno, Shigaku Ikeda

**Affiliations:** *From the Department of Dermatology, Juntendo University School of Medicine, Tokyo, Japan. E-mail: kharuna@juntendo.ac.jp*

**Figure 1:**
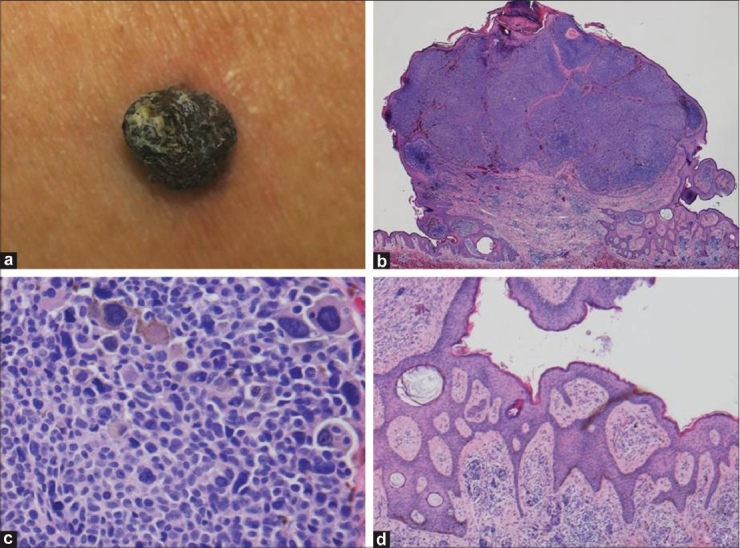
(a) A dark brown nodule with verrucous surface on the back, measuring approximately 1.0 cm; (b) Histopathological examination of the excised specimen revealed a pedunculated tumor with epidermal hyperplasia and irregular hyperkeratotic crusts (H and E, original magnification ×10); (c) Proliferation of highly atypical melanocytes of varying sizes was observed with numerous mitoses (H and E, original magnification ×400); (d) A reticulated structure with extension of epidermal protrusions and pseudo-horn cysts was observed on the pedicle of the tumor (H and E, original magnification ×100)

Sir,

On April 15, 2007, a 28-year-old Japanese male presented to our clinic for the evaluation of a nodule on his back [Figure 1a]. The lesion was approximately 1.0 cm in diameter and characterized by a uniformly dark–brown colour and hyperkeratotic verrucous surface. Examination with a magnifying glass showed the existence of milia-like cysts and comedo-like openings on the surface, meeting the dermoscopic criteria for seborrheic keratosis (SK).[1] Therefore, we excised the nodule with a 2.0 mm margin.

Pathologic examination of the specimen revealed a protuberant pedunculated tumor with a thickened stratum corneum with crusting in the upper part of the lesion. Large and highly atypical melanocytes formed alveolar-like structures immediately below the epidermis to the upper layer of the dermis [Figure 1b]. At a higher magnification, proliferation of highly atypical melanocytes of varying sizes with numerous mitoses was observed in tumor cell nests [Figure 1c]. The tumor cell nests were limited to the superficial layer of the dermis, and no infiltration into the deep dermis was observed. Many premelanosomes surrounding the nuclei of atypical melanocytes were positive for HMB-45 staining.

Interestingly, squamous papilloma-like features and laminated pseudocysts of horn were observed in several parts of the outer portion of the tumor [Figure 1d].

The patient was diagnosed as having nodular melanoma with a SK-like clinical presentation.[2–6] The Breslow thickness was 3.5 mm and the lesion was classified as pT3aN0M0, for the TNM stage, and stage IIA. As treatment, wide excision with a 3.0 cm surgical margin was performed. DAV-feron therapy was initiated as postoperative adjuvant chemotherapy. To date, his serum 5-S-CD levels have remained within normal limit. No evidence of metastasis has been observed on imaging studies, including CT, gallium, and IMP scintiscan.

Verrucous malignant melanoma (VMM) is a rare variant of melanoma first described in 1967.[2] Both clinically and histologically, it mimics SK.[2–6] Kuehnl-Petzoldt *et al*. have reported diagnosing VMM in 101 (9%) out of 1108 patients,[3] and Blessings *et al*. reported the condition in 20 (3.2%) out of 618 patients with melanoma.[6] Seventy-one percent of such melanomas are on the upper and lower extremities, but may occur on any anatomic site.[3]

Because melanoma is a nonepithelial skin cancer, it is of interest that the clinical features in the present case highly resembled those of SK. In previous literature,[7] intradermal and compound nevi have been described as showing hyperkeratosis, papillomatosis, horn cysts, and lace-like downward growth of epidermal strands. Though specific causative factors linking SK-like epidermal changes are still unknown, it is possible that both nevi and melanoma can release some epidermal cell growth factors, thereby inducing changes in the overlying epidermis.[7]

The present case highlights the clinical existence and features of such benign-looking melanomas.[8] They usually lack the characteristic signs of the “ABCD rule”. Therefore, Giacomel *et al*.[9] proposed the additional clinical features known as the *EFG rule*, that is elevated, firm skin lesions showing continuous growth, for diagnosing melanoma. Carbon dioxide laser removal of SK has become increasingly popular in many cosmetic clinics. Fewer routine histopathological evaluations may lead to an increase in the number of incorrect diagnoses. Finally, we emphasized the possibility of melanoma in the differential diagnosis and the importance of biopsy even in apparent SK cases.
